# Cardiac effects of ephedrine, norephedrine, mescaline, and 3,4-methylenedioxymethamphetamine (MDMA) in mouse and human atrial preparations

**DOI:** 10.1007/s00210-022-02315-2

**Published:** 2022-11-01

**Authors:** Joachim Neumann, Karyna Azatsian, Christian Höhm, Britt Hofmann, Ulrich Gergs

**Affiliations:** 1 Institute for Pharmacology and Toxicology, Medical Faculty, Martin Luther University Halle-Wittenberg, Magdeburger Str. 4, D-06097 Halle, Germany; 2 Department of Cardiac Surgery, Mid-German Heart Center, University Hospital Halle, D-06097 Halle, Germany

**Keywords:** Ephedrine, Norephedrine, 3,4-Methylenedioxymethamphetamine (MDMA), Mescaline, Inotropy, Chronotropy, Human atrium, Troponin inhibitor

## Abstract

The use of recreational drugs like ephedrine, norephedrine, 3,4-methylenedioxymethamphetamine (MDMA), and mescaline can lead to intoxication and, at worst, to death. One reason for a fatal course of intoxication with these drugs might lie in cardiac arrhythmias. To the best of our knowledge, their inotropic effects have not yet been studied in isolated human cardiac preparations. Therefore, we measured inotropic effects of the hallucinogenic drugs ephedrine, norephedrine, mescaline, and MDMA in isolated mouse left atrial (mLA) and right atrial (mRA) preparations as well as in human right atrial (hRA) preparations obtained during cardiac surgery. Under these experimental conditions, ephedrine, norephedrine, and MDMA increased force of contraction (mLA, hRA) and beating rate (mRA) in a time- and concentration-dependent way, starting at 1–3 µM but these drugs were less effective than isoprenaline. Mescaline alone or in the presence of phosphodiesterase inhibitors did not increase force in mLA or hRA. The positive inotropic effects of ephedrine, norephedrine, or MDMA were accompanied by increases in the rate of tension and relaxation and by shortening of time of relaxation and, moreover, by an augmented phosphorylation state of the inhibitory subunit of troponin in hRA. All effects were greatly attenuated by cocaine (10 µM) or propranolol (10 µM) treatment. In summary, the hallucinogenic drugs ephedrine, norephedrine, and MDMA, but not mescaline, increased force of contraction and increased protein phosphorylation presumably, in part, by a release of noradrenaline in isolated human atrial preparations and thus can be regarded as indirect sympathomimetic drugs in the human atrium.

**Supplementary information:**

The online version contains supplementary material available at 10.1007/s00210-022-02315-2.

## Introduction

**Fig. 1:**
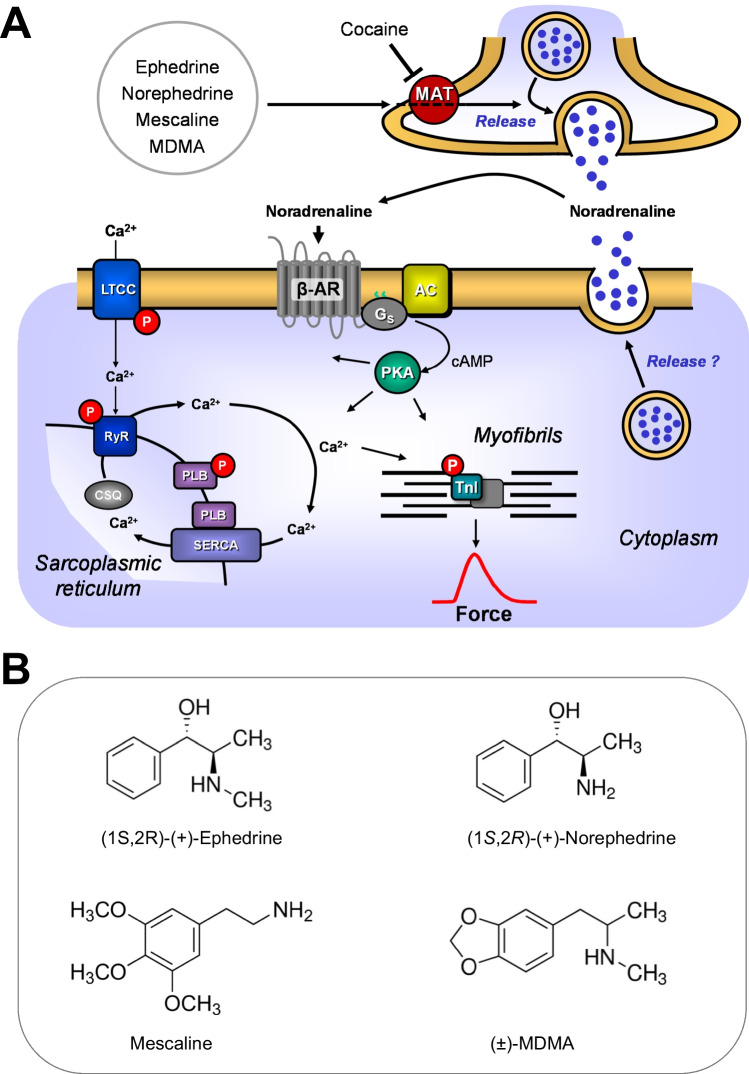
**A** Ca^2+^ enters the mammalian heart cell via the L-type Ca^2+^ channel (LTCC). This process can be enhanced by noradrenaline or isoprenaline via a cascade starting in the sarcolemma via stimulatory G-proteins (G_s_), elevates subsequent production of cAMP, and thereby activates cAMP-dependent protein kinase (PKA). PKA increases cardiac force generation and relaxation by increasing the phosphorylation state (P) of the L-type calcium channel (LTCC), of phospholamban (PLB), and of the inhibitory subunit of troponin (TnI). Trigger Ca^2+^ initiates release of Ca^2+^ from the sarcoplasmic reticulum via ryanodine receptors (RYR) into the cytosol. There, Ca^2+^ activates myofilaments and this activation leads to increased inotropy. In diastole, Ca^2+^ is taken up into the sarcoplasmic reticulum via a sarcoplasmic reticulum Ca^2+^-ATPase (SERCA), the activity of which is enhanced due to an increased phosphorylation state of PLB. Cocaine inhibits the activity of monoamine transporters (MAT). Ephedrine, norephedrine, MDMA, or mescaline might release noradrenaline from nerve terminals or cardiomyocytes but seem not to directly stimulate β-adrenoceptors. **B** Structural formulae of ephedrine, norephedrine, MDMA, and mescaline

The organic molecules (1R,2S)-2-methylamino-1-phenylpropane-1-ol (L-ephedrine), (1S,2R)-2-amino-1-phenyl-propane-1-ol (norephedrine; phenylpropanolamine), 3,4-methylenedioxymethamphetamine (MDMA; ecstasy), and 2-(3,4,5-trimethoxyphenyl)-ethyl-amine (mescaline) can be regarded as phenylethylamine derivatives (Fig. 1). As such, they can be interconverted by one or two chemical steps. They are structurally similar to 2-amino-1-(3,4-dihydroxyphenyl)-ethanol (noradrenaline) but in contrast to noradrenaline, they are often regarded as indirect sympathomimetics, because they may act mainly by release of noradrenaline from tissue or cells (Fig. 1) that then activates adrenoceptors. In contrast to noradrenaline, ephedrine, norephedrine, MDMA, and mescaline are not hydroxylated on the benzene ring and, therefore, they can be perorally applied because they are not extensively metabolized in the gastrointestinal tract. Nevertheless, they are to a certain extent, degraded to active metabolites or inactive metabolites (MDMA: (Carvalho et al. 2012); mescaline: (Dinis-Oliveira et al. 2019); ephedrine: (Sever et al. 1975); norephedrine: (Goodman 1980; Sinsheimer et al. 1973)).

Ephedrine was initially isolated from the plant *Ephedra edulis*. Ephedrine is an important drug, because it is on the World Health Organization (WHO) list of essential medicines (World Health Organization 2019). Ephedrine is sometimes used during anesthesia by obstetricians in the USA to raise blood pressure during childbirth (Ngan Kee and Khaw 2006; Shekelle et al. 2003; Xu et al. 2019). Ephedrine is also used to treat obesity, asthma, and narcolepsy (Shekelle et al. 2003). Likewise, ephedrine might be of high therapeutic value in some rare neurological diseases (Eirís-Puñal et al. 2020). Ephedrine is sometimes (mis-)used to improve performance in athletes (Miller 2004; Shekelle et al. 2003) and therefore found in dietary supplements for athletes (Miller 2004). To stop this misuse, ephedrine is on the list of prohibited substances of the world anti-doping agency (Docherty 2008; Docherty and Alsufyani 2021; World Anti-Doping Agency 2022).

The main side effects of ephedrine include tachycardia, hypertension, and hallucinations (Bolli 2008; Boroda and Akhter 2008). Ephedrine is thought to induce hallucinations by stimulation of 5-HT_2a_-receptors. Moreover, ephedrine increases alertness and deduces sleepiness by its direct or indirect action on adrenergic receptors in the brain, which probably explains the “recreational” (mis-)use of ephedrine. Ephedrine is traditionally contained in some over-the-counter drug mixtures against the symptoms of the common cold and is taken for that indication by patients often unbeknownst of their physicians: the attending physician is sometimes surprised why these patients present with hypertension (Bolli 2008). There are still clinical efforts under way to find new indications for ephedrine: one finds 92 clinical trials for ephedrine that have tested or test possible further clinical indications of ephedrine (at clinicaltrials.gov). Hence, it is relevant to understand human cardiac effects of ephedrine better.

Norephedrine (we have used here 1S,2R-( +)-norephedrine), which is a demethylated derivative of ephedrine (Fig. 1), is usually regarded as an indirect sympathomimetic agent. However, using Propadrine® (D,L-phenylpropanolamine, = racemic norephedrine), Trendelenburg’s group detected a positive chronotropic effect in guinea-pig right atrial preparations, about 50% which persisted (with a maximum at 66 µM) even in reserpinized preparations and argued that norephedrine exerted both direct and indirect sympathomimetic effects in the mammalian heart (TRENDELENBURG and CROUT 1964). Norephedrine could increase levels of cAMP in cells transfected with human β-adrenoceptors suggesting that norephedrine binds as an agonist at these receptors (Vansal and Feller 1999). Norephedrine was removed from the market in many countries because it was often misused by athletes and others (for the same reasons as ephedrine). In many cases, dietary supplements contain a synthetic racemic form of norephedrine (phenylpropanolamine (FDA 2000; Watson et al. 2010)). Norephedrine is still detected in illicitly marketed diet supplements for athletes. Using the search term “norephedrine,” one finds 56 clinical trials (at www.clinicaltrials.gov).

In general, MDMA can (Fig. 1) release noradrenaline (Rickli et al. 2016; Rothman et al. 2003). MDMA can also act as a direct agonist in vascular tissue on α_1,2_-adrenoceptors and 5-HT_2a_-receptors (review: (Al-Sahli et al. 2001; Docherty 2008)). After injection of 20 mg/kg MDMA, tachycardia in living rats ensued that was explained as a consequence of the rising of the core body temperature (known to be due to MDMA) but not stimulation of cardiac adrenoceptors (Gordon et al. 1991). Long-term (weeks or months) treatment of animals with MDMA altered the expression of hundreds of genes in gene chip analysis in rat hearts (Koczor et al. 2015). However, MDMA was also reported to bind to β-adrenoceptors (Battaglia et al. 1988). MDMA in therapeutic dosage (125 mg) in healthy volunteers, increased systolic and diastolic blood pressure, heart rate, body temperature, pupil size, and led to peak MDMA concentrations of 236 ng/ml (1.22 µM (Holze et al. 2020). MDMA is on the list of prohibited substances of the world anti-doping agency (Docherty 2008; World Anti-Doping Agency 2022). There is no accepted clinical indication for MDMA. However, there are 64 clinical trials on MDMA that test possible clinical indications of MDMA (at www.clinicaltrials.gov).

Mescaline (Fig. 1) is derived from a cactus growing at the border of Texas (USA) and Mexico (Goodman 1980; Seiler and Demisch 1971). It was used long before the Spaniards came to Mexico by locals as a hallucinogenic drug in religious ceremonies (reviewed in (Lewin 1888)). The hallucinogenic effects of mescaline are likewise explained by potent stimulatory actions of mescaline on brain 5-HT_2A_-receptors (Rickli et al. 2016). Mescaline was identified and named by Louis Lewin and purified by Heffter and synthesized by Späth (Heffter 1894; Lewin 1888; Späth 1919). Mescaline (100 µM and more) increased force of contraction in electrically driven rat atria and decreased the beating in rat atria in the organ bath (Siegl and Orzechowski 1977). However, as far as we could find out, effects of mescaline in isolated human cardiac muscle strips on force of contraction have not been previously published and are studied here for the first time. There is no accepted clinical indication for mescaline. However, there are four clinical trials that test possible clinical indications of mescaline (at www.clinicaltrials.gov). Some metabolites of mescaline may be hallucinogenic and therefore one might regard mescaline as a prodrug (Dinis-Oliveira et al. 2019). The systemic toxicity of mescaline is very low (Dinis-Oliveira et al. 2019).

To the best of our knowledge, inotropic effects of ephedrine, mescaline, or MDMA have never been studied in isolated human cardiac muscle strips and for norephedrine, only one study has been found (Kloth et al. 2017). This gap is closed by the present communication. To summarize, in this study, we tested the hypotheses that four structurally similar phenylalkylamines, namely ephedrine, norephedrine, MDMA, or mescaline increase contractility in isolated human atrial preparations and we studied mouse atrial preparations for comparison.

## Materials and methods

### Contractile studies on mouse atrial preparations

In brief, the right or left atrial preparations from 4- to 6-month-old CD-1 mice were isolated and mounted in organ baths as previously described (Boknik et al. 2019; Gergs et al. 2021; Neumann et al. 2021b). The bathing solution of the organ baths contained 119.8 mM NaCI, 5.4 mM KCI, 1.8 mM CaCl_2_, 1.05 mM MgCl_2_, 0.42 mM NaH_2_PO_4_, 22.6 mM NaHCO_3_, 0.05 mM Na_2_EDTA, 0.28 mM ascorbic acid, and 5.05 mM glucose. The solution was continuously gassed with 95% O_2_ and 5% CO_2_ and maintained at 37 °C and pH 7.4. Spontaneously beating right atrial preparations from mice were used to study any chronotropic effects.

### Contractile studies on human atrial preparations

The contractile studies on human preparations were done as described before (Boknik et al. 2019; Gergs et al. 2009, 2021). Human right atrial preparations were used with the same setup and buffer as described for mouse preparations (see above). The samples were obtained from male patients aged 67–82 years (mean ± SD: 73.1 ± 5.5 years; *n* = 14) undergoing bypass surgery. Drug therapy included metoprolol, furosemide, apixaban, and acetyl salicylic acid. The clinical state of the patients was quite different: some suffered from coronary heart disease without apparent alterations in atrial function; some showed paroxysmal atrial fibrillation that seemed to be confirmed, to our surprise, in the organ bath by episodes of spontaneously beating of the isolated trabeculae; some had a COPD that had deteriorated global systolic function translating into reduced potency of isoprenaline in the organ bath. Hence, clinical state varied and might be relevant for our results. Unfortunately, a correlation between patient characteristics and experimental results was not feasible because of the low number of patients together with these wide discrepancies between patients.

### Western blotting

The homogenization of the samples, protein measurements, electrophoresis, primary and secondary antibody incubation, and quantification were performed following our previously established protocols (Boknik et al. 2019; Gergs et al. 2021; Neumann et al. 2021a, 2021b). The following primary antibodies were used: anti-phosphorylated troponin inhibitor (P-TnI; #4004, Cell Signaling Technology Europe, Leiden, Netherlands) and anti-calsequestrin (CSQ) used as loading control (#ab3516, Abcam, Cambridge, UK).

### Data analysis

Data shown are means ± standard error of the mean. Statistical significance was estimated using analysis of variance (ANOVA) or Student’s *t*-test as appropriate. A *p*-value < 0.05 was considered to be significant. The software Prism 5.0 (GraphPad Software, San Diego, CA, USA) was used for statistical analysis and creation of graphs.

### Drugs and materials

The drugs isoprenaline ((-)-isoproterenol ( +)-bitartrate salt), L-ephedrine (1R,2S-2-methylamino-1-phenylpropane-1-ol), ( +)-norephedrine (1S,2R-2-amino-1-phenyl-propane-1-ol), 3,4-methylenedioxy-methamphetamine (MDMA, ecstasy), and ( ±)-propranolol hydrochloride were purchased from Sigma-Aldrich (Steinheim, Germany). Mescaline (2-(3,4,5-trimethoxyphenyl)ethanamine) was from Cayman Chemical Company (Ann Arbor, MI, USA) distributed by the Biomol Company in Hamburg, Germany. All other chemicals were of the highest purity grade commercially available. Deionized water was used throughout the experiments. Stock solutions were prepared fresh daily.

## Results

### Studies in isolated left atrial preparations from mice

**Fig. 2:**
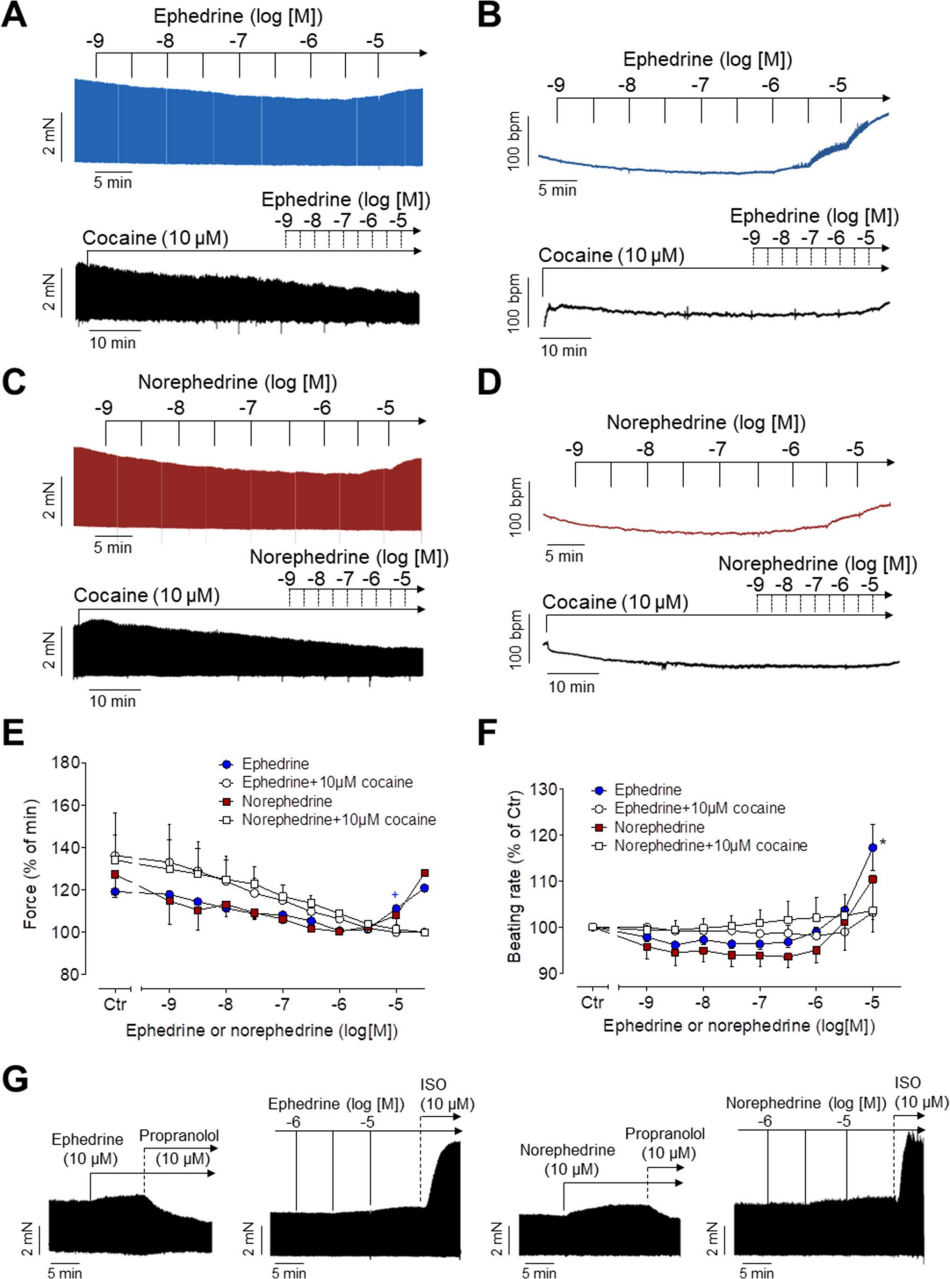
Inotropic and chronotropic effects of ephedrine and norephedrine. **A**–**D** Original recordings: ephedrine (**A**, **B**) and norephedrine (**C**, **D**) exert a concentration- and time-dependent positive inotropic effect in isolated electrically driven (1 Hz) left atrial preparations (**A**, **C**) and a positive chronotropic effect in isolated spontaneously beating right atrial preparations (**B**, **D**) of mice. The inotropic and chronotropic effects of ephedrine and norephedrine were antagonized by cocaine (10 µM). Horizontal bars: time axis (ms). Vertical bars: developed tension in milli-Newton (mN) or beating rate in beats per minute (bpm). These data are summarized in **E** and **F** for force of contraction and beating rate, respectively. Ordinates: force in % of minimum force (**E**) or beating rate in % of Ctr (**F**). Basal values (= control values) were 3.11 ± 0.48 mN (ephedrine) and 2.93 ± 0.41 mN (norephedrine) for the force of contraction and 404.6 ± 12.1 bpm (ephedrine) and 380.6 ± 15.6 bpm (norephedrine) for the beating rates. *N* = 3–4 (*N* = 2 for cocaine treatment). Ctr: pre-drug value before drug addition. Abscissae: decadic logarithm of the concentration of ephedrine or norephedrine in the organ bath. **G** Original recordings demonstrating the effect of propranolol (10 µM) and isoprenaline (ISO, 10 µM) on force of contraction of left atrial preparations applied in presence of 10 µM ephedrine (left side) or norephedrine (right side). *p < 0.05 versus Ctr; ^+^*p* < 0.05 versus minimum

**Fig. 3:**
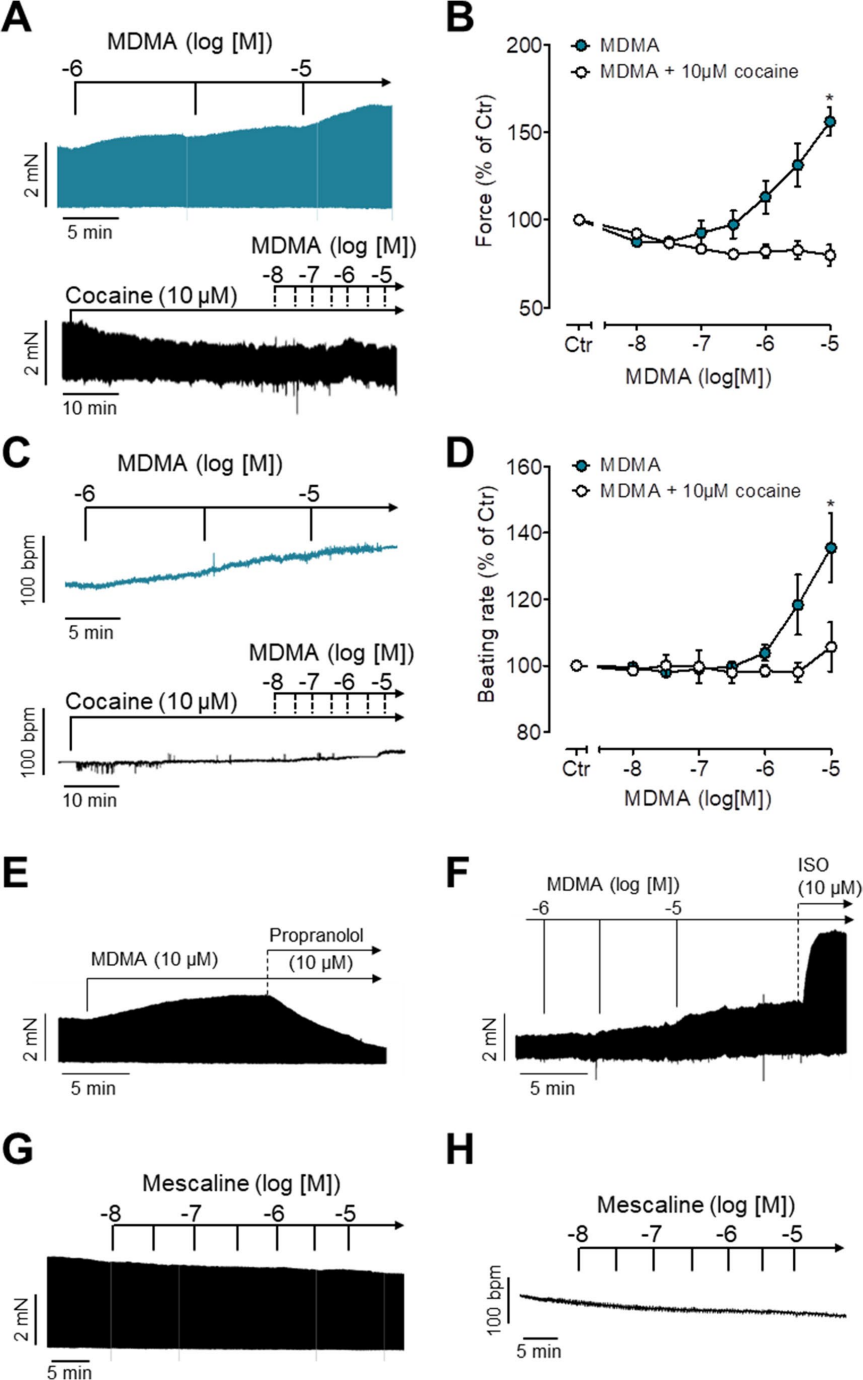
Inotropic and chronotropic effects of MDMA and mescaline. **A**, **C** Original recordings: MDMA exerts a concentration- and time-dependent positive inotropic effect in isolated electrically driven (1 Hz) left atrial preparations (**A**) and a positive chronotropic effect in isolated spontaneously beating right atrial preparations (**C**) of mice. The inotropic and chronotropic effects of MDMA were antagonized by cocaine (10 µM). Horizontal bars: time axis (ms). Vertical bars: developed tension in milli-Newton (mN) or beating rate in beats per minute (bpm). These data are summarized in **B** and **D** for force of contraction and beating rate, respectively. Ordinates: force in % of Ctr (**B**) or beating rate in % of Ctr (**D**). Basal values (= control values) were 3.1 ± 0.31 mN for the force of contraction and 404.9 ± 15 bpm for the beating rate. *N* = 3–4. Ctr: pre-drug value before drug addition. Abscissae: decadic logarithm of the concentration of MDMA in the organ bath. **E**, **F** Original recordings demonstrating the effect of propranolol (10 µM) (**E**) and isoprenaline (ISO, 10 µM) (**F**) on force of contraction of left atrial preparations applied in the presence of 10 µM MDMA. **G**, **H** Mescaline failed to increase force of contraction (**G**) or beating rate (**H**) in mouse atrial preparations. *p < 0.05 versus Ctr; ^#^*p* < 0.05 versus cocaine

It is apparent from original recordings that ephedrine (Fig. 2), norephedrine (Fig. 2), and MDMA (Fig. 3) raised force of contraction in a concentration- and time-dependent manner in isolated electrically stimulated left atrial preparations from mice. These data are summarized in Figs. 2E and 3B. The time course of the increase in force of contraction after addition of ephedrine, norephedrine, and MDMA was visibly slower than effects of isoprenaline consistent with different signal transduction mechanisms. In contrast, mescaline failed to increase force of contraction (Fig. 3), while the atrial preparations were still responsive to 1 µM isoprenaline (data not shown) confirming that the β-adrenoceptors in these muscles were working. The positive inotropic effects of ephedrine, norephedrine, and MDMA were abrogated after pre-treatment of atria with 10 µM cocaine (Figs. 2A, C and 3A, bottom). Moreover, the positive inotropic effects of ephedrine, norephedrine, and MDMA were eliminated by additionally applied 10 µM propranolol (Figs. 2G and 3E). Subsequently, additionally applied 10 µM isoprenaline again raised force of contraction. The maximum inotropic effect of 10 µM ephedrine, norephedrine, and MDMA was about half of that of isoprenaline (Figs. 2G and 3F). This indicates that ephedrine, norephedrine, and MDMA are less potent and less effective than isoprenaline to raise force of contraction.

The efficacy but not the potency of ephedrine to increase force of contraction in a second application could be increased by pre-treatment of atrial preparations from mice with the phosphodiesterase inhibitor rolipram (0.1 µM) (data not shown).

### Studies in isolated right atrial preparations from mice

As seen in original tracings, ephedrine (Fig. 2), norephedrine (Fig. 2), and MDMA (Fig. 3) increased the heart rate in isolated spontaneously beating right atrial preparations from mice in a concentration- and time-dependent manner. These data are summarized in Figs. 2F and 3D. Ephedrine, norephedrine, and MDMA (10 µM each) were less effective and potent than 10 µM isoprenaline to increase the beating rate in mRA (data not shown). As seen in original tracings, in the additional presence of 10 µM cocaine, ephedrine (Fig. 2B, bottom), norephedrine (Fig. 2D, bottom), and MDMA (Fig. 3C, bottom) failed to increase the beating rate but subsequently isoprenaline 10 µM was effective to elevate beating rate indicating that the samples were responsive to stimulation of β-adrenoceptors (data not shown). The positive chronotropic effects of ephedrine, norephedrine, or MDMA were reversed by additionally applied propranolol suggesting the effects were due to stimulation of β-adrenoceptors (data not shown). In contrast, mescaline failed to increase the beating rate (Fig. 3H).

### Contractile studies in human atrial preparations

**Fig. 4:**
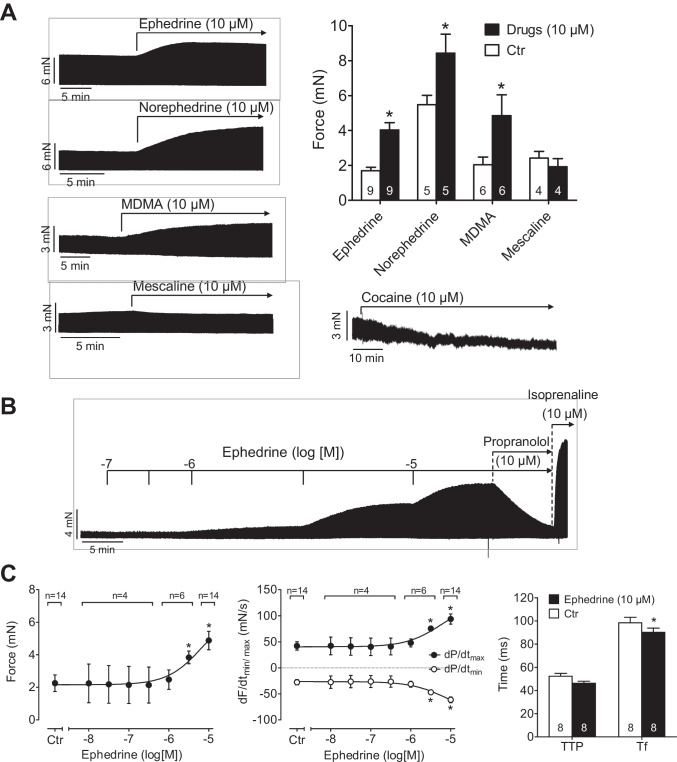
Effects of ephedrine, norephedrine, MDMA, and mescaline on force of contraction in human atrial preparations. **A** Original recordings of a non-cumulative application of ephedrine, norephedrine, MDMA, mescaline, and cocaine as control (10 µM each) to isolated electrically driven (1 Hz) human right atrial preparations. The data are summarized in the bar diagram. **B** Original recording of a cumulative application of ephedrine. Where indicated, propranolol (10 µM) and isoprenaline (10 µM) were added without washout of previously applied drugs. Horizontal bars: time axis (min). Vertical bars: developed tension in milli-Newton (mN). The data for cumulatively applied ephedrine are summarized in **C**: force of contraction (left), maximum rate of tension development (dF/dtmax) and of relaxation (dF/dtmin) (middle), time to peak tension (TTP), and time of relaxation (Tf) (right). The numbers of preparations are given in the graphs. *p < 0.05 versus Ctr

We compared cumulative application and non-cumulative application of ephedrine, norephedrine, MDMA, and mescaline. To find out whether these compounds act at all in human atrial preparations, in a first set of experiments, we only applied 10 µM of each drug (Fig. 4A). We noted that ephedrine, norephedrine, and MDMA increased force of contraction in isolated electrically stimulated right atrial preparations (Fig. 4A). These positive inotropic effects were accompanied by increased rates of tension development and shortened time to peak tension. Moreover, these effects led to shortened time of relaxation and faster rate of relaxation suggesting the involvement of β-adrenoceptors acting via cAMP and increased phosphorylation state of the troponin inhibitor (compare scheme in Fig. 1). However, the inotropic effects took more time (10 to 20 min) to reach the maximum than the positive inotropic effects of isoprenaline (which plateaued within 2 min) on the very same muscle preparation (Fig. 4B), suggesting different signal transduction mechanisms. Moreover, the effect of 10 µM ephedrine in the presence of 1 µM of the phosphodiesterase III inhibitor cilostamide amounted to about 25% of the effect of 10 µM isoprenaline (data not shown), indicating that ephedrine may be less effective than isoprenaline at least up to a concentration of 10 µM, the highest concentration tested here. Furthermore, the positive inotropic effects of 10 µM ephedrine, 10 µM norephedrine, and 10 µM MDMA could be blocked by 10 µM propranolol (data not shown).

Next, we studied cumulatively applied ephedrine, norephedrine, MDMA, or mescaline (10 nM until 10 µM). We noted a positive inotropic effect of ephedrine (Fig. 4B), norephedrine (data not shown), and MDMA (data not shown). These positive inotropic effects were smaller than the positive inotropic effect of 10 µM isoprenaline (Fig. 4B for ephedrine) and could be blocked by 10 µM propranolol (Fig. 4B for ephedrine). Indeed, any inotropic effects could be blocked by previously given 10 µM cocaine (a control incubation is shown in Fig. 4A) or 10 µM propranolol (data not shown). Moreover, we noted that MDMA alone increased force of contraction concentration-dependently; this effect started above 1 µM MDMA (data not shown). In contrast to MDMA, ephedrine or norephedrine, cumulatively applied, often failed to raise force of contraction. However, in the same trabeculae, in the presence of the phosphodiesterase III inhibitor cilostamide (1 µM), both ephedrine and norephedrine were able to elevate force of contraction (data not shown).

The situation was different for mescaline: neither a single application of mescaline (10 µM, Fig. 4A) nor a cumulative concentration–response curve of mescaline (10 nM to 10 µM, data not shown) nor in the additional presence of 1 µM cilostamide (data not shown), elicited a positive inotropic effect in separate human atrial preparations that responded to MDMA. Moreover, time to peak tension and time of relaxation, maximum rate of tension development, and maximum rate of relaxation remained unchanged in the presence of increasing concentrations of mescaline. This is relevant, because prolonged contraction time can occur without changes in maximum force of contraction (e.g., with omecamtiv: (Dashwood et al. 2021)).

### Studies on protein phosphorylation in human atrial preparations

**Fig. 5:**
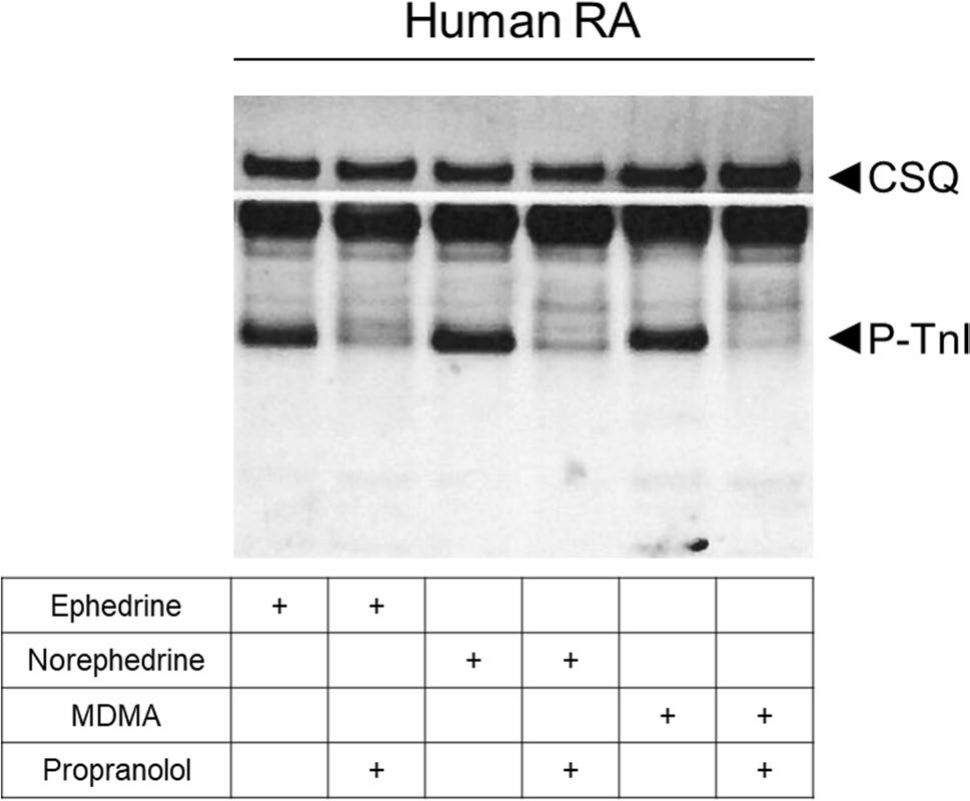
Ephedrine, norephedrine, and MDMA increase phosphorylation in isolated human atrial preparations. Typical Western blots demonstrate the effect of 10 µM ephedrine, norephedrine, or MDMA on troponin inhibitor (TnI) phosphorylation in isolated electrically stimulated (1 Hz) human right atrial preparations (RA). The phosphorylation of TnI was inhibited by application of 10 µM propranolol. As a loading control, we assessed the protein expression of calsequestrin (CSQ) by cutting the lanes of the blot and incubating the lower and upper halves with different primary antibodies

Fittingly, in contracting human atrial preparations, 10 µM ephedrine or 10 µM norephedrine or 10 µM MDMA alone increased the phosphorylation state of the inhibitory subunit of troponin (TnI) compared to samples with additionally applied 10 µM propranolol (Fig. 5). As loading control for cardiac preparations, the protein expression of calsequestrin (CSQ, see also Fig. 1) was studied on the same membranes.

## Discussion

To the best of our knowledge, evidence for a stimulatory action of ephedrine, norephedrine, and MDMA in isolated mouse atrial preparations has not been presented before. The main new result of the present work, in our eyes, is, however, that ephedrine and MDMA exert positive inotropic effects in human atrial preparations. For norephedrine, a positive inotropic effect in human atrial preparations has been reported recently (Kloth et al. 2017). Likewise, the lack of positive inotropic effect of mescaline in isolated mouse atrial preparations and human atrial preparations has, as far as we know, not been reported before.

Moreover, we could show that in isolated mouse and human atrial preparations, ephedrine, norephedrine, and MDMA but not mescaline can raise the phosphorylation state of TnI. It is known for a long time that TnI plays a significant role in the positive inotropic response to β-adrenergic stimulation (Layland et al. 2004). Increased phosphorylation of TnI can explain, at least in part, why ephedrine, norephedrine, and MDMA reduce the time of relaxation and increase the rate of tension relaxation in atrial preparations from mice and humans: phosphorylated TnI reduces the Ca^2+^ sensitivity of the myofilaments and increases the crossbridge cycling rate. Together with an increased rate of sarcoplasmic reticulum Ca^2+^ uptake due to phosphorylation of phospholamban, less Ca^2+^ binds to the myofilaments and myofilaments relax faster (Layland et al. 2004; Takimoto et al. 2004).

The lack of action of mescaline might be explained with the observation that mescaline neither stimulates adrenoceptors and nor inhibits noradrenaline transporters (Rickli et al. 2016). For ephedrine, the situation may be slightly more difficult. It is probably species dependent whether ephedrine is a direct or indirect sympathomimetic drug or both. In a series of seminal papers, Ullrich Trendelenburg observed the following: ephedrine exerted concentration-dependent positive chronotropic effects (maximum at 60 µM) in spontaneously beating isolated guinea-pig right atrial preparations (TRENDELENBURG et al. 1963; TRENDELENBURG 1966; TRENDELENBURG and CROUT 1964). These positive chronotropic effects of ephedrine were attenuated but not abrogated when they pretreated the living guinea pigs with reserpine 24 h before the contraction experiments. These reserpine-resistant positive chronotropic effects of ephedrine were regarded as β-adrenoceptor mediated. In other words, ephedrine exerted, convincingly, direct and indirect effects via β-adrenoceptors in the mammalian heart (TRENDELENBURG et al. 1963; TRENDELENBURG 1966; TRENDELENBURG and CROUT 1964). Comparable data in mouse and human cardiac atrium are currently lacking. In disagreement with the functional data of Trendelenburg, ephedrine has been claimed not to bind directly to β-adrenoceptors, using radioactive ligands (Rothman et al. 2003). However, ephedrine could bind to human α_2_-adrenoceptors, but only with low affinity (K_i_-values around 5 µM (Rothman et al. 2003)). Moreover, others, in vitro, detected that ephedrine could increase cAMP concentrations in cells transfected with human β-adrenoceptors suggesting that ephedrine can, indeed, functionally bind as an agonist at these receptors (Vansal and Feller 1999). In addition, or alternatively, ephedrine can release noradrenaline in some isolated neuronal tissues and thus can act as an indirect sympathomimetic drug (Rothman et al. 2003) (Fig. 1). Later work convincingly reported on a direct stimulatory action of ephedrine on α-adrenoceptors in rat vessels or in living mice (Liles et al. 2006, 2007). There are clinical data that ephedrine acts via β_2_-adrenoceptors: in gynecological patients, ephedrine’s efficacy to increase blood pressure correlated with the mutations in β_2_-adrenoceptors (review: (Rao et al. 2019)). However, our data can be reconciled with their findings when one argues that we measured directly inotropy in the muscle whereas they measured in vivo a complex interaction of central and peripheral effects with intact compensatory mechanisms being obvious. Hence, direct effects of ephedrine on adrenoceptors in the atrium cannot be ruled out.

But it is unlikely that under our experimental conditions, ephedrine, norephedrine, and MDMA directly stimulate β-adrenoceptors and thus increase force of contraction and the beating rate: this conclusion is based on the observation that the contractile effects of ephedrine, norephedrine, and MDMA were absent in the present of cocaine. In line with previous studies in other experimental animals (review: (Docherty 2008)), we would argue here that cocaine inhibits the noradrenaline transporter that would normally allow ephedrine, norephedrine, and MDMA to pass outer cell membranes and then release noradrenaline from intracellular pools (Fig. 1).

In human cardiomyocytes produced from stem cells, ephedrine starting at 0.5 µM increased the spontaneous beating rate (Calvert et al. 2015). Force of contraction was not measured (Calvert et al. 2015). It is unclear whether these cells are of an atrial or ventricular phenotype (Calvert et al. 2015). Clinical data on donors are missing and it is unusual for non-diseased human cardiac cells not originating from the sinus node to beat spontaneously. However, these data might indicate that ephedrine can release noradrenaline from cardiomyocytes. It has been reported before that cardiomyocytes themselves possibly can form and can contain catecholamines (Ebert et al. 2008). Apart from that, intrinsic cardiac adrenergic cell types that have been demonstrated to be present in rodent and human hearts may be the origin of the released noradrenaline (Huang et al. 1996; Saygili et al. 2011). Under our experimental conditions, we cannot discriminate whether ephedrine releases noradrenaline from cardiomyocytes or from ganglia or from intrinsic cardiac adrenergic cells or from all (Fig. 1). This point should be the subject of further studies for instance using isolated human atrial cardiomyocytes in primary culture but this is beyond the scope of the present study.

The clinical relevance is that “recreational drugs” like ephedrine, norephedrine, MDMA, and mescaline can lead to intoxication and death. One reason for a fatal course of intoxication with ephedrine, norephedrine, and MDMA might lie in cardiac arrhythmias. One manifestation of cardiac arrhythmias is tachycardia. This we saw with ephedrine, norephedrine, and MDMA in mouse right atrial preparations. In vivo coronary constriction due to serotonin acting on 5-HT_2a_ receptors and released noradrenaline acting on alpha-adrenergic receptors in the vessel wall might contribute to cardiac arrhythmias. Moreover, our data suggest that intoxication to ephedrine, norephedrine, and MDMA can be treated by propranolol as far as their action as noradrenaline releasing agents in the heart are concerned. A popular over the counter medication in Germany is a syrup that contains 6.17 mg ephedrine in 30 ml that accounts for an about 1.2 mM solution. One can predict that this syrup can lead to µM concentrations in the blood, which is in the range where cardiac side effects from our data in human atrial preparations are predicted. It is deplorable that such medications are still on the market.

One can ask what are the highest concentrations of ephedrine or norephedrine reached in the human body. Some information might be gained from intoxications, which should represent the upper limits of concentrations in humans. Hence, concentrations of up to 0.40 mg/kg (= 2.6 µM) in blood for ephedrine and 0.40 mg/kg (= 2.4 µM) in blood for norepinephrine have been reported (Nedahl et al. 2019). In intoxications, levels of MDMA as high as 6 µM and 70 µM have been reported (Carvalho et al. 2012; Peters et al. 2003). For mescaline, plasma concentrations as high as 14.8 mg/l (66 µM) have been reported (Reynolds and Jindrich 1985).

In humans, in the course of intoxications, MDMA has been suggested to cause death by cardiac arrhythmias (Carvalho et al. 2012). Moreover, in humans, MDMA could cause deadly myocardial infarction (Carvalho et al. 2012). In a clinical trial, MDMA led to sinus tachycardia and an increase in systolic and diastolic blood pressure (Lester et al. 2000). In contrast to the present findings, others noted no measurable inotropic effects when up to 40 mg/kg per minute of MDMA was given to volunteers (Lester et al. 2000). One might argue that either their detection methods (transthoracic echocardiography) was less sensitive than ours or that they obtained lower levels of MDMA in the heart (Lester et al. 2000). Most likely their methods were not sensitive enough: the noted tachycardia and this alone by the so-called Bowditch Treppe phenomenon increases inotropy in the human ventricle (Bombardini et al. 2003; Mulieri et al. 1992). Hence, our data shed new light on the topic and clarify that MDMA in principle can have a positive inotropic effect in the human heart, at least in the human atrium. It has been hypothesized by others that MDMA might lead to the production of free radicals in the human heart, leading to altered Ca^2+^ homeostasis and finally contraction band necrosis (Carvalho et al. 2012).

Hence, these are concentrations where increases in contractility gain significance under our experimental conditions. By extrapolation to the human heart in patients, one might be tempted to speculate that direct contractile effects of ephedrine and norephedrine can occur but at the upper limit of the therapeutic window where in some persons already intoxications occur. On the other hand, one could argue that tachycardia in users of ephedrine and norephedrine might be due also to direct effects on the heart and not solely to indirect effects initiated in the brain via the sympathetic nerve system and that such tachycardia should be treatable by application of β-adrenoceptor blockers. In this case, β-adrenoceptor blockers would be useful due to their direct action on cardiac of β-adrenoceptors and not alone due to their action on the central nerve system. However, based on the assumption that indirect sympathomimetic drugs would increase noradrenaline levels in the venous effluent of the coronary veins or in the wall of arterioles that are responsible for the resistance in the coronaries, the question arises: would the application of β-adrenoceptor blockers lead to a vasospasm? It is clear that noradrenaline can stimulate α_1_ and β_1_ and β_2_-adrenoceptors. Functional data argue that noradrenaline dilates the human coronaries via β_2_-adrenoceptors (Sun et al. 2002). In the same study, the authors have investigated the role of 10 µM propranolol (the concentration we used here) and found that β_2_ action of propranolol attenuates the vasodilatory action of noradrenaline but does not unveil a vasoconstrictor action of noradrenaline (Sun et al. 2002). Moreover, a case report in which pseudoephedrine ingestion caused electrocardiogram changes of acute myocardial ischemia, which was relieved by use of the β_1_-selective adrenoceptor blocker metoprolol, at least partially supported our hypothesis (Akay and Ozdemir 2008). Sometimes, ephedrine is used without prescription in liquids, solid, and ointment to treat symptoms of common cold. Our data might be interpreted as not supporting the use of these over the counter medicines because direct undesired effects on the heart cannot be ruled out.

In summary, we presented evidence for ephedrine, norephedrine, and MDMA but not for the chemically related mescaline to act as indirect sympathomimetic agents in the isolated human atrium by measurement of inotropic effects. We hypothesize from our data that drugs that block β-adrenoceptors (e.g., propranolol) should be tried in cases of intoxication by these drugs. However, clinical studies will be needed to test our hypothesis that cardiac side effects of ephedrine can be treated with β-adrenoceptor blockers like propranolol.

## Supplementary information

Below is the link to the electronic supplementary material.

Supplementary file1 (PDF 238 KB)

## Data Availability

The data of this study are available from the corresponding author upon reasonable request.
