# Supplementary material for: Cardiac effects of ephedrine, norephedrine, mescaline, and 3,4-methylenedioxymethamphetamine (MDMA) in mouse and human atrial preparations
Source: Naunyn Schmiedebergs Arch Pharmacol. 2022 Nov 1;396(2):275–87. doi: 10.1007/s00210-022-02315-2 (PMC9831963; doi:10.1007/s00210-022-02315-2)
Supplement: Supplementary file 1 — Supplementary file1 (PDF 238 KB) [file 210_2022_2315_MOESM1_ESM.pdf]

Complete original Western blots. Please note that the blotting membrane was cut before antibody incubation for economically motivated reasons and to detect the loading control on the very same blot. Moreover, because of species-dependent differences in the amino acid composition of CSQ and TnI, the apparent molecular weight of mouse and human CSQ and TnI differs.

MW	−C	E	E+P	NE	NE+P	M	M+P	+C	+Cb	E	E+P	NE	NE+P	M	M+P	M	M+P	+C	MW

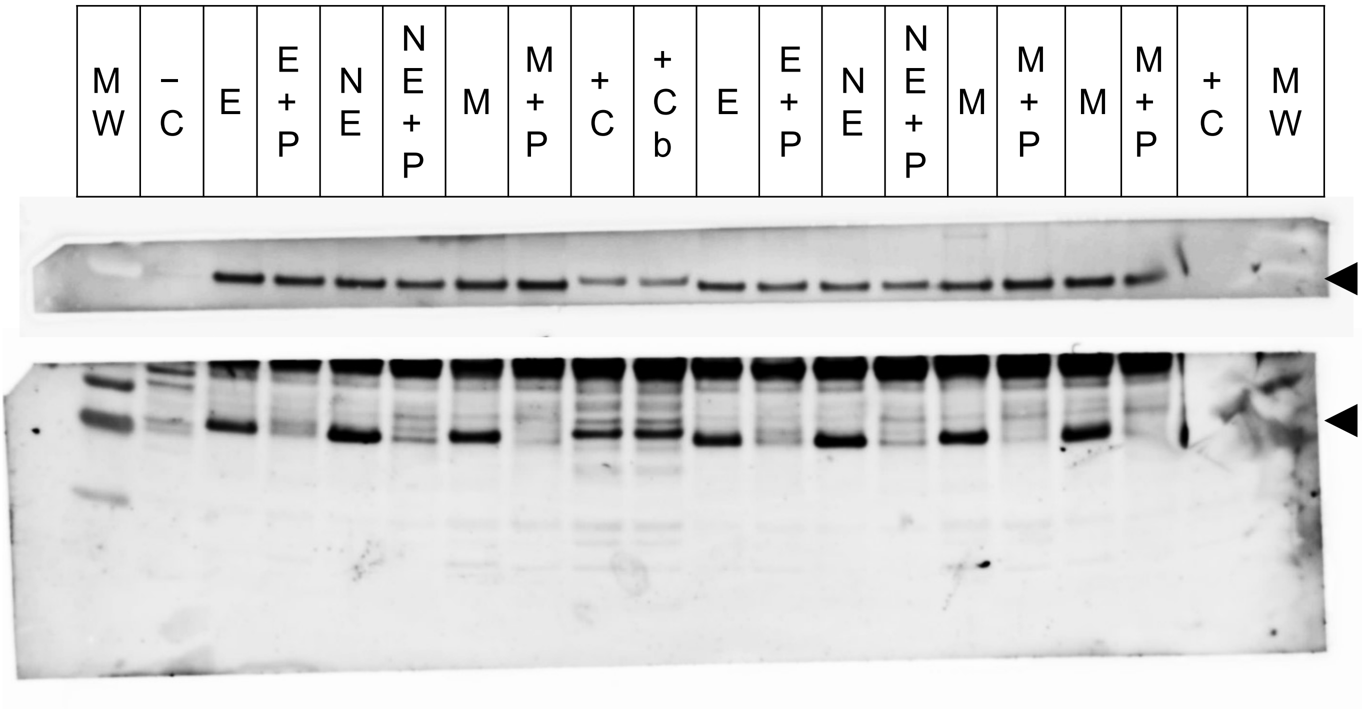

Abbreviations:
CSQ, calsequestrin (used as cardio myocyte-specific loading control)
P-TnI, phosphorylated troponin inhibitor
MW, molecular weight marker
-C, negative control (mouse sample)
+C, positive control (mouse sample)
+Cb, positive control boiled (mouse sample)
E, ephedrine (10 μM) (human sample)
E+P, ephedrine (10 μM)+propranolol (10 μM) (human sample)
NE, norephedrine (10 μM) (human sample)
NE+P, norephedrine (10 μM)+propranolol (10 μM) (human sample)
M, MDMA (10 μM) (human sample)
M+P, MDMA (10 μM)+propranolol (10 μM) (human sample)
